# Barley *HvPAPhy_a* as transgene provides high and stable phytase activities in mature barley straw and in grains

**DOI:** 10.1111/pbi.12636

**Published:** 2016-11-01

**Authors:** Inger Bæksted Holme, Giuseppe Dionisio, Claus Krogh Madsen, Henrik Brinch‐Pedersen

**Affiliations:** ^1^ Department of Molecular Biology and GeneticsFaculty of Science and TechnologyResearch Centre FlakkebjergAarhus UniversitySlagelseDenmark

**Keywords:** barley, genetic transformation, grain, HvPAPhy_a phytase, straw

## Abstract

The phytase purple acid phosphatase (HvPAPhy_a) expressed during barley seed development was evaluated as transgene for overexpression in barley. The phytase was expressed constitutively driven by the cauliflower mosaic virus 35S‐promoter, and the phytase activity was measured in the mature grains, the green leaves and in the dry mature vegetative plant parts left after harvest of the grains. The T_2_‐generation of *HvPAPhy_a* transformed barley showed phytase activity increases up to 19‐fold (29 000 phytase units (FTU) per kg in mature grains). Moreover, also in green leaves and mature dry straw, phytase activities were increased significantly by 110‐fold (52 000 FTU/kg) and 57‐fold (51 000 FTU/kg), respectively. The *HvPAPhy_a*‐transformed barley plants with high phytase activities possess triple potential utilities for the improvement of phosphate bioavailability. First of all, the utilization of the mature grains as feed to increase the release of bio‐available phosphate and minerals bound to the phytate of the grains; secondly, the utilization of the powdered straw either directly or phytase extracted hereof as a supplement to high phytate feed or food; and finally, the use of the stubble to be ploughed into the soil for mobilizing phytate‐bound phosphate for plant growth.

## Introduction

Phytases (myo‐inositol hexakisphosphate 3‐ and 6‐phosphohydrolase; EC 3.1.3.8 and EC 3.1.3.26) are phosphatases that can hydrolyze phytic acid (InsP_6_, myo‐inositol‐(1,2,3,4,5,6)‐hexakisphosphate), the most important phosphorous (P) storage compound in plant seeds, representing 40%–80% of total seed P (Eeckhout and De Paepe, 1994; Lott, 1984). In its degradation of phytic acid, phytases play a fundamental biological role, as it ensures bioavailable phosphate needed for regular progression of germination. However, also from applied perspectives, phytase in plants has a range of potentials. First of all, high phytase activity in feed and food is most wanted because the digestive tracts of nonruminants possess negligible phytase activity. Moreover, the activity provided by food and feedstuffs is often nonexisting or insufficient for efficient hydrolysis of the InsP6, which is therefore excreted via the manure along with chelated essential minerals such as zinc, calcium and iron (Brinch‐Pedersen *et al*., 2014; Rostami and Giri, 2013).

Increment of the phytase activities in seeds through genetic transformation has been accomplished in several crops including wheat, rice, maize, soybean and canola (Brinch‐Pedersen *et al*., 2000, 2003, 2006; Chen *et al*., 2013; Denbow *et al*., 1998; Gao *et al*., 2007; Hong *et al*., 2004; Lucca *et al*., 2001; Peng *et al*., 2006). Focus has not been on using the plants own phytases but on using microbial enzymes belonging to the group of histidine acid phosphatases. Basically, the phytases used are the same as the ones used in industrial production of feed enzymes. In addition to increasing seed phytase activity, microbial phytase has also been expressed in green vegetative tissues of tobacco, alfalfa and potato. The purpose of this was to use green leaves for extraction of phytase (Ullah *et al*., 1999, 2002, 2003). Accumulation of functional phytase in mature dead vegetative plant parts left after seeds harvest has so far not been reported but constitutes a potential valuable alternative for phytase production. With a yearly estimated production of mature barley straw, on around 3 tons per hectare or 55 kg straw per 100 kg grain (Statistic Denmark; http://www.dst.dk/en) valorization of this tissue is highly attractive.

Scientific initiatives in recent years have led to a substantially increased knowledge base on the complement of cereal phytases and paved the way for using the plants own enzymes for improving phytase activity in seeds and vegetative tissues. Cereal grains contain HAP phytases (the multiple inositol polyphosphate phosphatase (MINPP) phytase) but the bulk of activity can be attributed to phytases belonging to a different group of phosphatases, the purple acid phosphatase (Dionisio *et al*., 2007, 2011). In Triticeae tribe cereals, purple acid phosphatase phytase (*PAPhy*) genes generally consist of a set of paralogues, *PAPhy_a* and *PAPhy_b*. The promoters share a conserved core, but the *PAPhy_a* promoter have acquired a novel *cis*‐acting regulatory element for expression during grain filling while the *PAPhy_b* promoter has maintained the archaic function and drives expression during germination. PAPhy_a accumulates in the aleurone layer and scutellum during grain development and contributes to the mature grain phytase activity (MGPA) of Triticeae cereals that is barley, wheat and rye (Madsen *et al*., 2013). Non‐Triticeae cereals for example maize and rice only has one *PAPhy* gene which is predominantly expressed during germination (Dionisio *et al*., 2011). In agreement with this, their mature grains have very low phytase activity. The cereal PAPhy pH optimum is 5.5 ± 0.14, and the temperature profile is broad with optimum at 55 °C ± 1.8 °C (both values for TaPAPhy_a1) (Dionisio *et al*., 2011). These biochemical parameters are in comparable range to the commercial phytase products the *Aspergillus niger‐*based phytase, Natuphos and the *Perniphora lycii* phytase, Ronozyme NP (Menezes‐Blackburn *et al*., 2015).

The purpose of this study is to evaluate the transgenic potential of the barley grain phytase gene *HvPAPhy_a* constitutively expressed by the 35S‐promoter for (I) increased mature grain phytase activity in barley, (II) for accumulation of functional enzyme in green leaves and (III) for its accumulation in mature plant tissues as potential side product from barley production. A series of transgenic barley plants constitutively expressing the *HvPAPhy_a* gene were analysed, and highly elevated phytase activities were achieved in mature grains, green leaves and also in mature straw. HvPAPhy_a accumulation in vegetative tissues were monitored by nanoLC‐MS, and high levels of easy extractable phytase activities were recorded in a series of mature vegetative tissues of *HvPAPhy_a* transformed barley.

## Results

### MGPA in primary transformants

**Figure 1:**
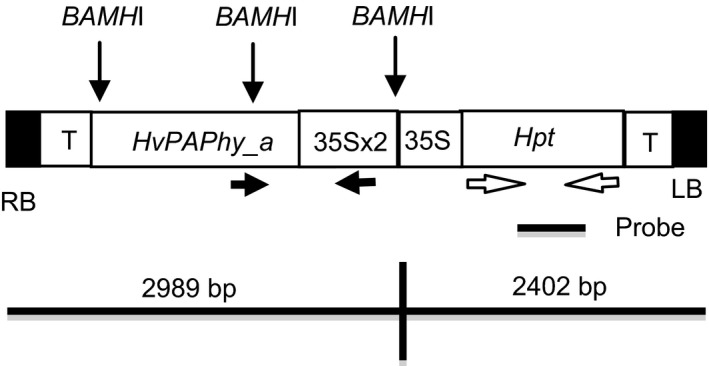
Diagram of the T‐DNA of the *35S:PAPhy_a* vector. The *Bam* *H*I restriction sites are indicated. The primers utilized for PCR analysis are shown with arrows, and the position of the *Hpt* probe employed for Southern blotting is indicated. RB: right border; LB: left border; T: 35S‐terminator.

We infected 200 embryos with Agrobacterium strain AGL0 containing the construct *35S:PAPhy_a* (Figure 1). This leads to 20 primary transformants (T_0_). PCR analysis for the hygromycin resistance gene was utilized to verify that the plants contained the T‐DNA of the vectors (data not shown). Seventeen of the *35S:PAPhy_a* transformants were fertile.

**Figure 2:**
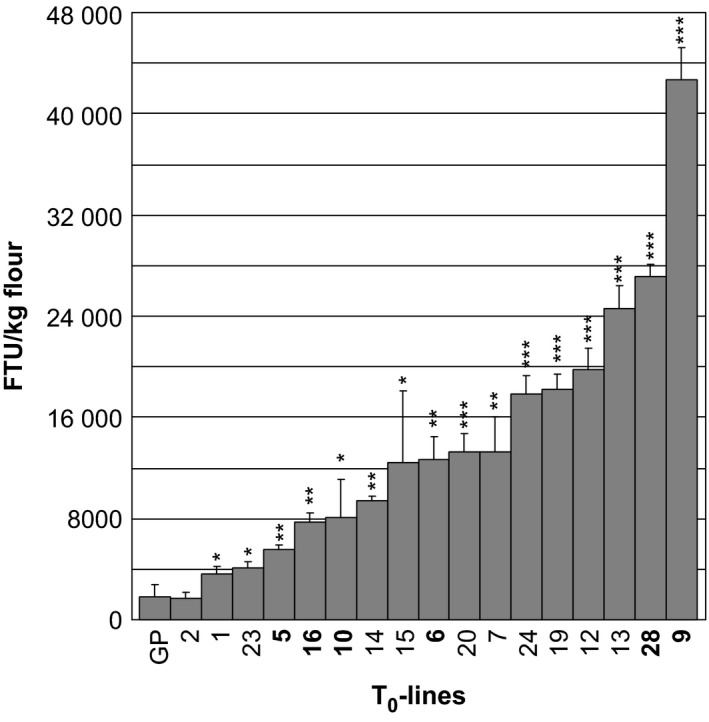
Mature grain phytase activity of T_0_‐plants transformed with *35S:PAPhy_a*. GP: nontransformed control. Bars represent standard deviations (SD). Asterisks indicate significance levels: * significant different from nontransformed control (GP) at the 5% level, **significant different from nontransformed control (GP) at the 1% level, ***significant different from nontransformed control (GP) at the 1‰ level. The plant numbers that are further investigated in the T_1_‐generation are in bold.

As T_0_ grains are segregating for the transgene, mature grain phytase activity (MGPA) measurements were performed on pools of 20–25 grains from each T_0_‐plant. With exception of transformant 2 which were not significantly different from the control MGPA (1863 phytase units (FTU) per kg flour), all other *35S:PAPhy_a*‐transformed plant showed significantly higher MGPAs, ranging from 3672 to 42 700 FTU per kg flour (Figure 2).

### Progeny from primary transformants

Three T_0_‐plants in the lower MGPA range (≤8000 FTU/kg) and three plants in the higher MGPA range (>12 000 FTU/kg) were selected for further investigations. PCR analysis was utilized to identify progeny positive for the transgene. To distinguish from the endogenous *PAPhy_a*, primer combinations were used where forward priming in the 35S promoter was combined with reverse priming in the *PAPhy_a* coding area (see Figure 1 for indication of primer sites). Inserts were detected in 11 of 13 progenies (Figure S1).

**Figure 3:**
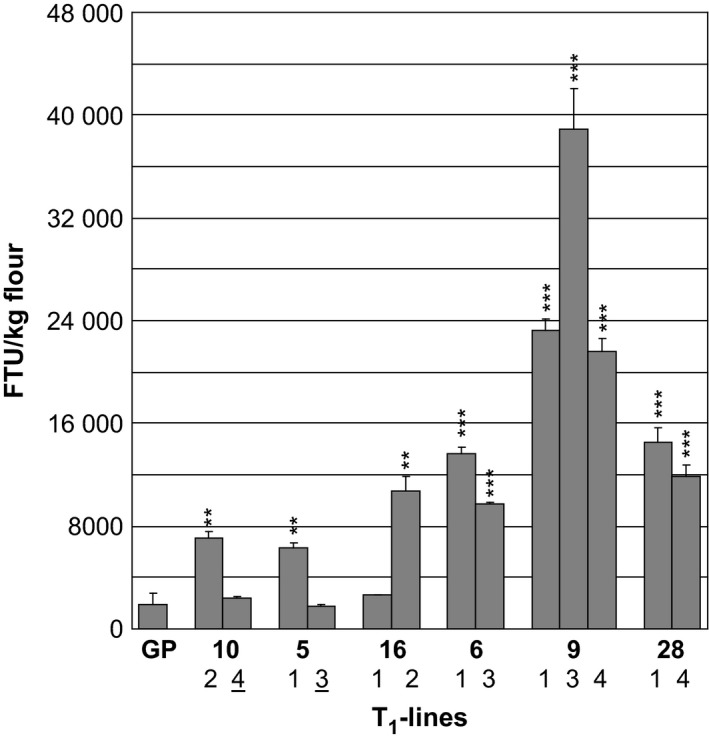
Mature grain phytase activity of T_1_‐plants transformed with *35S:PAPhy_a*. GP: nontransformed control. The numbers of the T_1_ progeny correspond to numbers on Figure 2 and Figure S1. Bars represent SD. Asterisks indicate significance levels: for explanation see legend for Figure 2. The numbers underlined indicate the T_1_‐plants where no transgene was detected in the PCR analysis (Figure S1).

With the exception of plant 16.1 (with an MGPA of 2697 FTU), progeny containing the *35S:PAPhy_a* transgene, all had significantly increased MGPA as compared with the nontransformed control (Figure 3). The two progenies with no *35S:PAPhy_a* inserts had nonsignificant MGPAs as their activities were within the range of the nontransformed control.

The differences in MGPA between the *35S:PAPhy_a* plants could be influenced by integration number effects of the transgenes. Therefore, the number of integration sites in progeny of plants with varying MGPAs was investigated by Southern blotting (Figure S2). For the *35S:PAPhy_a* plants 5.1 (6280 FTU/kg), 6.1 (13 680 FTU/kg), 28.4 (11 913 FTU/kg) and 9.4 (21 551 FTU/kg), one, two, two and three *PAPhy_a* integrations, respectively, were revealed by the Southern blot. This supports a relationship between integration site number and the MGPA. However, a direct linear comparison between integration site number and phytase activity is difficult as T_1_‐plants can be either heterozygous for the insert with the insert in only one of the two homologous chromosomes or homozygous for the insert with an insert in both homologous chromosomes. We were not able to distinguish these in the Southern blots.

### PAPhy_a protein in green leaves

The presence of the PAPhy_a protein in green flag leaves of T_1_‐plant *35S:PAPhy_a‐28.4* and a nontransformed control were analysed by nanoLC‐MS/MS. Flag leaves were collected just before the appearance of the awns. Peptide mapping of PAPhy_a is shown in Figure S3. The nanoLC‐MS/MS analysis showed that PAPhy_a was present at 1225 ng/g fresh weight (FW) among the leaf soluble proteins of *35S:PAPhy_a‐28.4* whereas none of them were present among the leaf soluble proteins of the nontransformed control (Table 1). As a control, three common enzymes present in the flag leaves were also quantified using the same procedure and were found to be present in similar amounts in both plants investigated (Table 1). Furthermore, the quantitative values of all the proteins investigated were very similar over the triplicate biological harvests and triplicate LC‐MS runs and identifications with standard deviation between 4.5% and 6% between triplicates.

**Table 1:** Relative and absolute quantification of *HvPAPhy_a* (in bold), *HvMINPPII_a* and some representative barley leaf soluble enzymes in nontransformed barley (control) and *35S:PAPhy_a* (T_1_‐plant 28.4). Data are presented as a single LC‐MS run identification. However, standard deviations over triplicate experiments were below 6% for all the data reported

Acces. no.	Description	MW Da	Control			*35S:PAPhy_a* (28.4)		
			Score	fmol	ng/g FW	Score	fmol	ng/g FW
P00924	Eno1_yeast	4664	164	150	–	150	150	–
**C4PKL2**	**PAPhy_a**	**60 297**	**0**	**0**	**0**	**24**	**24**	**1225**
A0FHA8	MINPPII_a	58 145	0	0	0	0	0	0
P26517	GAPDH1	36 513	60	55	1345	53	53	1285
F2CR16	Aldolase A	37 896	59	54	1359	38	38	952
K7X0F7	GS1	38 774	9.9	9.1	234	9.7	9.7	251

Eno1 yeast: yeast enolase 1, added and utilized as relative quantification control; GAPDH1: Glyceraldehyde‐3‐phosphate dehydrogenase (cytosolic); Aldolase A: Fructose‐bisphosphate aldolase; GS1: Glutamine synthetase 1.

### Phytase activity in green and dry mature vegetative plant parts of *35S:PAPhy_a* transgenic plants

**Figure 4:**
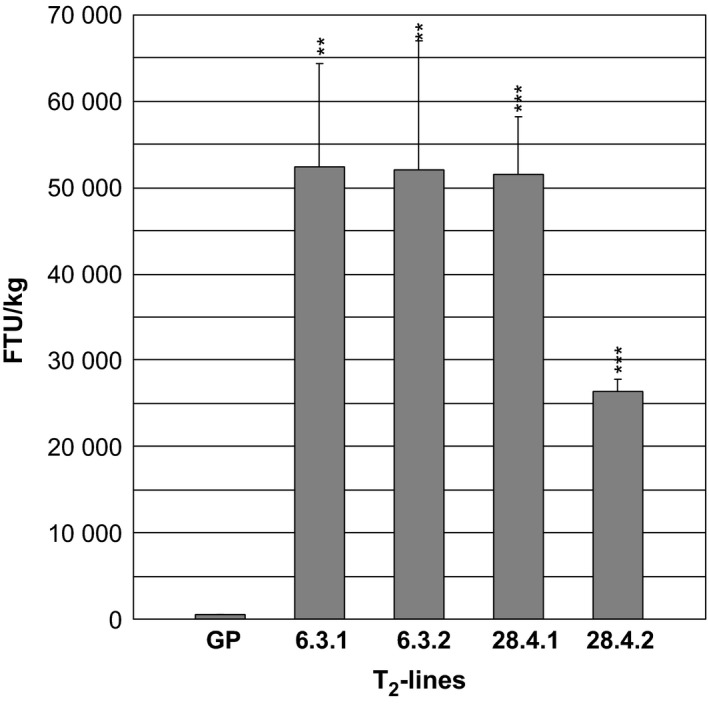
Phytase activity in green leaves of T_2_‐*35S:PAPhy_a* plants. Bars represent SD. Asterisks indicate significance levels: for explanation see legend for Figure 2.

The phytase activity was measured in green flag leaves excised just before the appearance of the awns of two progenies from T_1_‐plant *35S:PAPhy_a*‐28.4 and in two progenies of T_1_‐plant *35S:PAPhy_a*‐6.3 (Figure 4). The phytase activities in the leaves were very high ranging from 26 405 FTU/kg for plant *35S:PAPhy_a*‐28.4.2 to 52 330 FTU/kg for plant *35S:PAPhy_a*‐6.3.1.

**Figure 5:**
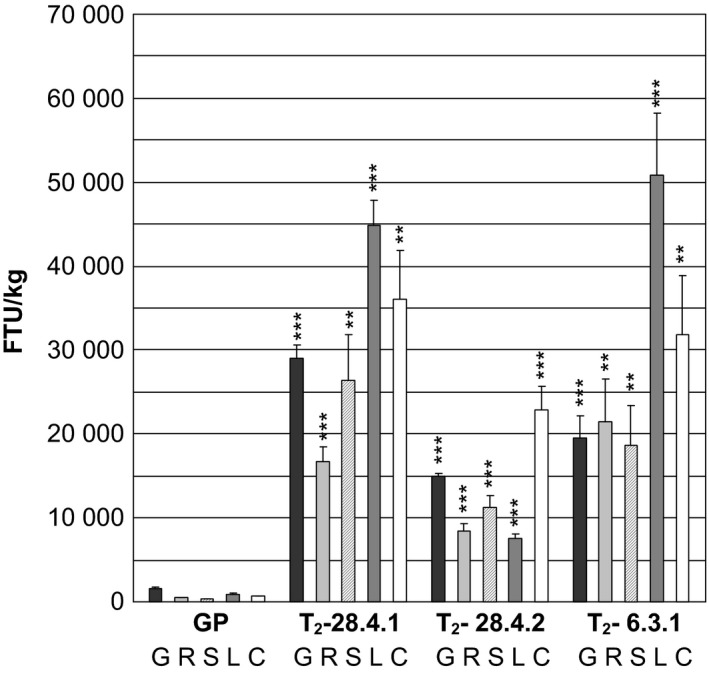
Phytase activity in freshly harvested dry mature grains (G), rachis (R), stems (S), leaves (L) and chaff and awns (C) of a nontransformed control (GP) and T_2_‐*35S:PAPhy_a* plants. Bars represent SD. Asterisks indicate significance levels: for explanation see legend for Figure 2.

Just after harvest of the mature plants, the level of preserved functional phytases was also assayed in the mature dry leaves, stems, rachis, chaff and awns in three of these T_2_‐plants (*35S:PAPhy_a*‐28.4.1, *35S:PAPhy_a‐*28.4.2, *35S:PAPhy_a*‐6.3.1). Initial experiments measuring free phosphate in the dry vegetative material protein extracts by the molybdovanadate method before incubation revealed that the extracts from nontransformed plants had a free phosphate content. Thus, in the subsequent activity assays, the free phosphate in the dry vegetative material was subtracted from the assay result. As shown in Figure 5, the phytase activities in the leaves, stem, rachis and chaff and awns of the nontransformed controls are low (320–890 FTU/kg). In contrast, the phytase activities in *35S:PAPhy_a*‐28.4.2 leaves, stem, rachis and chaff and awns accounted for 7621, 11 181, 8357 and 22 816 FTU/kg dry tissue, respectively. Even higher phytase activities were measured in leaves, stem, rachis and chaff and awns of *35S:PAPhy_a*‐28.4.1 with 44 922, 26 384, 16 794 and 22 816 FTU/kg dry tissue, respectively and in *35S:PAPhy_a*‐6.3.3.1 with 50 850, 18 707, 21 465 and 31 751 FTU/kg dry tissue, respectively (Figure 5). Overall, these results show that high levels of PAPhy can be synthesized in green vegetative barley tissue in a fully functional form and that most of this remains intact during senescence and can be found in freshly harvested mature dry tissue.

### Phytase activity in 3‐year‐old stored dry vegetative plant parts of *35S:PAPhy_a* transgenic plants

The level of preserved functional phytase was assayed in mature rachis, chaff and awns from two T_2_‐progenies from *35S:PAPhy_a*‐28.4 and one T_2_‐progeny plant from *35S:PAPhy_a*‐6.3 that had been stored for 3 years.

**Figure 6:**
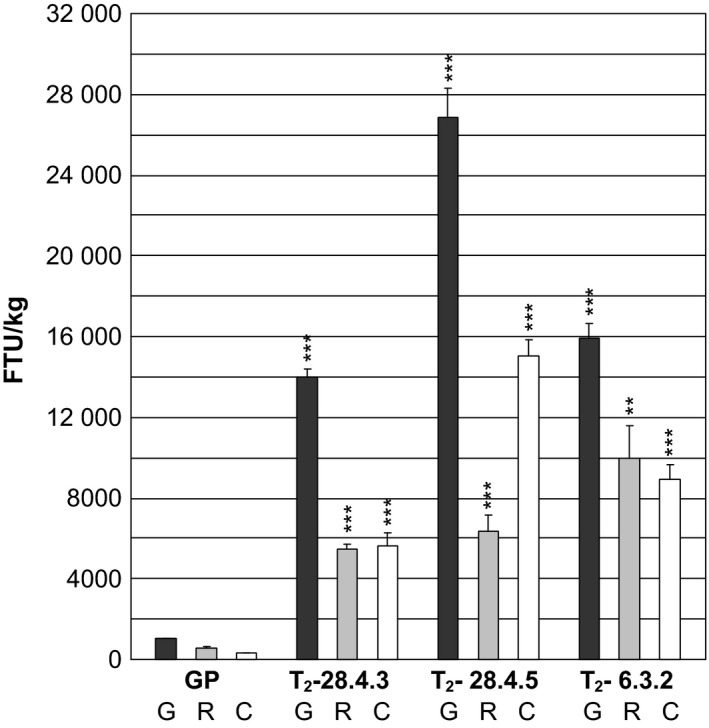
Phytase activity in 3‐year‐old stored dry grains (G), rachis (R) and chaff and awns (C) of a nontransformed control (GP) and T_2_‐*35S:PAPhy_a* plants. Bars represent SD. Asterisks indicate significance levels: for explanation see legend for Figure 2.

As shown in Figure 6, the phytase activities in *35S:PAPhy_a*‐28.4.3 rachis and chaff and awns both accounted for 4800 FTU/kg dry tissue, respectively. Even higher phytase activities were measured in rachis and chaff and awns of *35S:PAPhy_a*‐28.4.5 with 6100 and 14 800 FTU/kg dry tissue, respectively and in *35S:PAPhy_a*‐6.3.2 with 10 000 and 9000 FTU/kg dry tissue, respectively. Overall, these results show that a high percentage of the PAPhy_a initially synthesized in the vegetative barley tissue can be stored in a fully functional form in mature dry tissue for minimum 3 years.

## Discussion

In the present study, we have evaluated *in planta* the *HvPAPhy_a* phytase as transgene in barley. The results clearly demonstrate that 35S:HvPAPhy_a‐transformed plants reached highly elevated phytase activities in both grain and green leaves. Yet, the most important finding of this study is that we also observed high phytase activities in mature dead vegetative tissue not only just after harvest but also in the corresponding tissue stored for 3 years.

The increases in mature grain phytase activity (MGPA) were stably inherited to the T_1_‐ and T_2_‐transgenic plants that were selected for further investigations. The MGPAs found in some of the barley plants constitutively expressing the *HvPAPhy_a* are very high. However, similar or higher MGPAs have been reported in other transgenic crops expressing microbial‐derived phytases (reviewed by Gontia *et al*., 2012). Also, large differences in MPGA were observed between the barley plants constitutively expressing the *HvPAPhy_a* gene. This is most likely caused by position effects of the transgenes and/or differences in the number of transgene integrations. Southern blots of four selected T_1_‐plants might indicate a possible correlation between the number of *35S:PAPhy_a* transgene integrations and MGPA. We have previously found a positive correlation between phytase activity and plants being heterozygous or homozygous for the *HvPAPhy_a* cisgene (the genomic clone of *HvPAPhy_a* including the native promoter and terminator of the gene) (Holme *et al*., 2012). Here, plants heterozygous (one insert) for the *HvPAPhy_a* cisgene resulted in a twofold increase while plants homozygous (two inserts) for the cisgene resulted in a 2.7‐fold increase in MGPA. Although we cannot distinguish between heterozygous and homozygous inserts in the Southern blots of this study, the present study shows that one insert of the *35S:PAPhy_a* transgene can lead to a higher increase (3.5‐fold) in MGPA than one or two inserts of the cisgene. The results therefore indicate that utilization of the constitutive promoter causes higher increases in MGPA than the use of the native promoter which controls expression in only the aleurone layer and scutellum and not in the entire endosperm.

Likewise, expression of the *35S:PAPhy_a* transgene in vegetative tissue was expected due to the constitutive promoter. This was confirmed by the nanoLC‐MS/MS investigation of young flag leaves from the T_1_‐plants *35S:PAPhy_a*‐28.4. Here, the PAPhy_a enzyme was detected at a concentration of 1225 ng/g FW while the PAPhy_a phytase could not be detected in leaves of the nontransformed control. The concentration of *35S:PAPhy_a*‐28.4 in leaves was almost similar to the glycolytic enzymes glycealdehyde‐3‐phosphate dehydrogenase and fructose‐bisphosphate aldolase which are some of the most abundant enzymes in cells (Table 1).

We subsequently measured the phytase activities in the green leaves of progeny from the *35S:PAPhy_a*‐28.4 plant. In these green leaves, we found very high increases in phytase activities of up to 110‐fold. High increases in phytase activity of green vegetative tissue ranging from fivefold to 273‐fold have previously been reported in different plants expressing microbial phytases including tobacco, alfalfa, potato and rice (Hamada *et al*., 2005; Ullah *et al*., 1999, 2002, 2003). In these studies, the purpose was to purify the phytase or use the green tissue directly for feed before the start of senescence.

However, in the present study, we also focused on phytase activity in mature dry dead vegetative tissue left after harvest of the grains. The high phytase activities that we found in milled mature dry vegetative tissue of *35S:PAPhy_a* plants just after harvest of the grains clearly show that preformed HvPAPhy_a phytase could be stored in the straw, leaves, rachis, chaff and awns with little degradation and remobilized during senescence. As both microbial and plant grain phytases are functional in highly proteolytically active environments, special properties of the enzymes may allow the enzyme also to be very resistant to proteolysis during senescence in leaves and stems. This is supported by a previous study where a heterologous *A. niger* phytase accumulating in tobacco leaves was found to be present at much higher levels than other proteins in senescent leaves (Verwoerd *et al*., 1995). In that study, they compared the degradation of two different heterologous proteins that is the *Aspergillus* phytase and the human serum albumin and found that only the phytase was present in high amounts in senescent leaves. Therefore, the authors suggested that the phytase protein is more resistant to degradation during senescence due to the heavy glycosylation and folding of the protein in a way that makes it resistant to degradation (Verwoerd *et al*., 1995). Similarly, a heterologous yeast phytase transgene with the phytase derived from *Schwanniomyces occidentalis* showed high activity levels in young leaves of transformed rice of up to 10 600 FTU/kg FW. Most importantly, the leaf extracts utilized for phytase assays could be stored for at least 4 weeks with minimal loss of phytase activity whereas the concentration of other soluble proteins in these extracts showed strong degradation after only 1 week of storage (Hamada *et al*., 2005). These results indicate that phytase enzymes in general possess a native resistance towards proteolytic degradation. Phytase activities in mature dry vegetative transgenic tissue have, however, not been previously reported in any crop. Additionally, our results show that the HvPAPhy_a enzyme is not only protected from degradation during senescence but also retains much of the phytase activity in the mature vegetative parts during storage at room temperature for 3 years.

**Figure 7:**
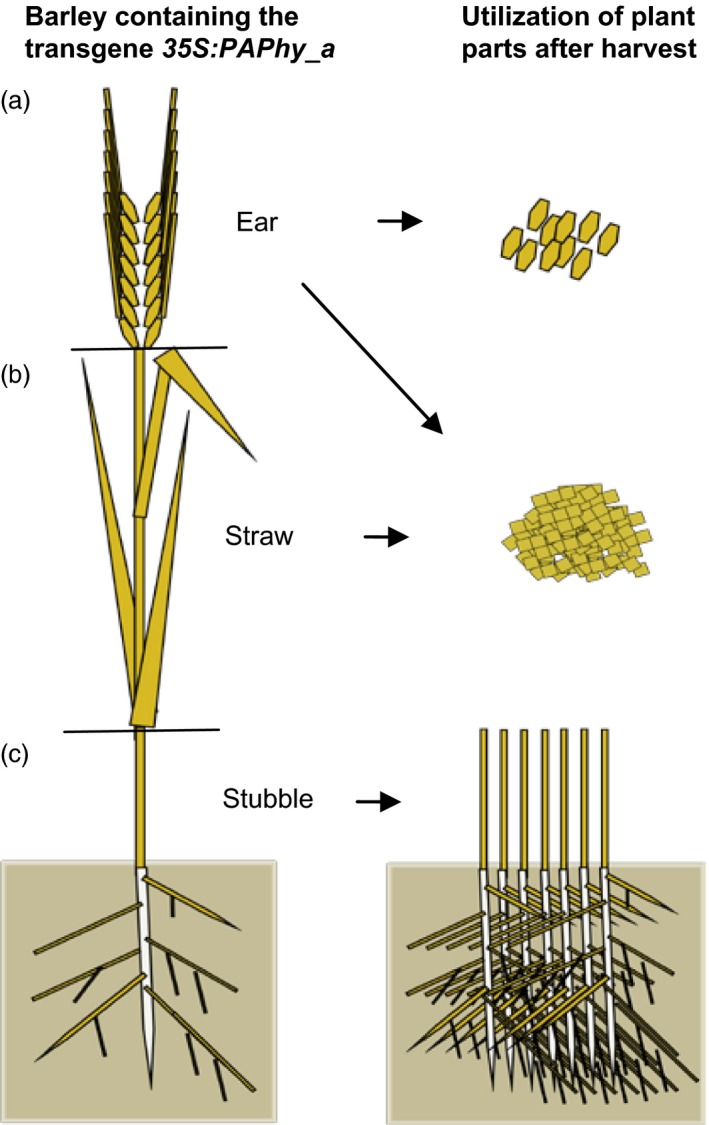
Illustration of the possible potentials of growing barley containing the transgene *35S:PAPhy_a*. (a) Grains with high phytase activities can be utilized directly for food and feed to increase the release of bioavailable phosphate and minerals bound to the phytate of the grains. (b) Powdered straw, rachis, chaff and awns with high phytase activities can be used either directly or the phytase can be extracted and used as supplements to high phytate feed and food. (c) The stubble can be ploughed into the soil to mobilize phytate‐bound phosphate for plant growth.

The presence of such high preformed phytase levels in the mature dry vegetative material of the *35S:PAPhy_a*‐plants adds further potentials to the utilization of these transgenic plants than using the high phytase grains for feed and food (Figure 7). The straw from *35S:PAPhy_a* plants can be collected during harvest of the grains, milled to a very fine powder and added to feed or food as an extra supplement of phytase activity either directly or as extracts hereof as the phytase can very easily be water extracted from the tissue. This could be a great substitute for commercially microbial phytase that is often added to the feed of monogastric animals. Taken into account that supplement of microbial phytase to the feed at a concentration of 1500 FTU/kg makes about 60% of the phosphate bound in phytate bioavailable (Kerr *et al*., 2010), then addition of extracts from only 50 g of nonstored powder from plant *35S:PAPhy_a* 6.3.1 to one kilo of the feed would have the same effect on phosphate bioavailability. Similarly extracts from the fine powder could be supplemented to high phytate human food to increase the nutritional value of the food not only with respect to phosphate but also the other essential minerals bound to phytate that is zinc, calcium and iron (Brinch‐Pedersen *et al*., 2007). As importantly, the phytase activity in the stubble left after harvest of fields with these plants may, if the stubble is ploughed down into the soil, contribute to the soil fertility by a faster release of phosphate and other minerals bound to phytate present in the soil.

As previously mentioned, PAPhy_a phytases are only present in Triticeae cereals. Among these, we chose to overexpress the *HvPAPhy_a* gene in barley. Barley is considered a model species for the Triticeae cereals as it is a well‐characterized diploid cereal with the genome almost fully sequenced (The International Barley Genome Sequencing Consortium 2012). Barley also has a number of molecular and genetic techniques are available, including a relatively efficient *Agrobacterium*‐mediated transformation system (Mrizova *et al*., 2014). Particularly, barley has the advantage in biosafety aspects of being a self‐pollinating species with for many cultivars; a closed type of flowering and outcrossings with other barley plants or related species are extremely rare (Ritala *et al*., 2002). Despite the reduced environmental risk of transgenic barley as compared to many other crops, chances of acceptance for deliberate release of any transgenic crop in EU are very low. Also, the costs and timely procedures for obtaining approval of GM crops for cultivation are a major impediment (Holme *et al*., 2013). Still, the *35S:PAPhy_a* plants possess the advantage that the HvPAPhy_a enzyme is a barley endogenous enzyme, for which it is already known that there are no allergenic or toxic risks. Hence, the magnitude of risk assessment data requirements for food and feed safety of the *35S:PAPhy_a* plants can be reduced. As regulations in USA is less stringent, approval of the *35S:PAPhy_a* plants would probably be somewhat faster and less expensive than in the EU. But as a consequence of the high prices of GM approval worldwide, increases in bioavailable phosphate from feed are still more economically achieved by a simple supplement of microbial phytases to the feed. However, the great potentials of phytase production in the straw can only be accomplished by a transgenic approach.

In conclusion, the present study demonstrates for the first time the transgenic potentials of the *HvPAPhy_a* gene in barley. Transgenic expression of the gene led to highly increased phytase levels in grains, in green leaves and as a novel discovery also in dead mature straw. High phytase levels were detected in mature vegetative tissues after up to 3 years of storage. The current results demonstrate that *HvPAPhy_a* is a potent phytase in transgenic barley and that it has multiple applications with potential implications for nutrient bioavailability and environment.

## Experimental procedures

### Vector designs

The *PAPhy_a* cDNA was cloned and characterized by Dionisio *et al*., 2011;. *PAPhy_a* was PCR amplified from the cDNA clone using USER primers (Hebelstrup *et al*., 2010; Nour‐Eldin *et al*., 2006). The primers were Hv_PAPHy_a_user_fw GGCTTAAUATGCCAAGCAACAACATCAA and Hv_PAPhy_a_user_rv GGTTTAAUTTACGGACCGTGTGCGGGC. The PCR reaction was carried out using PfuTurbo Cx Hotstart DNA polymerase (Stratagene) according to the manufacturer's instructions. Subsequently, each amplicon was cloned using USER^™^ cloning into the pCambia130035Su vector (Nour‐Eldin *et al*., 2006). In the resulting vector, *35S:PAPhy_a*, the *PAPhy_a* open reading frames are under control of the cauliflower mosaic virus 35‐S promoter and terminator (Figure 1). The vector was transformed into *Agrobacterium* strain AGL0 using the freeze/thaw method and selected on medium with 50 mg/L kanamycin and 25 mg/L rifampicin.

### Transformation

The spring barley cultivar Golden Promise was grown in growth cabinets with a 15/10 °C day/night temperature regime and 16 h light period at a light intensity of 350 μE/m^2^/s. Immature embryos isolated 12–14 days after pollination were used for *Agrobacterium* transformation following the procedure of Bartlett *et al*. (2008) with the exception that 500 μm acetosyringone was included in the cocultivation medium (Hensel *et al*., 2008) and 30 mg/L hygromycin was used instead of 50 mg/L in the transition, regeneration and rooting medium and that 0.1 mg/L benzylaminopurine was added to the regeneration medium. Regenerated T_0_‐plants and their progenies were grown in a greenhouse.

### PCR analysis of T_0_‐plants, T_1_‐progeny and T_2_‐progeny

The transgene in the primary transformants (T_0_) was verified by PCR analysis using primers for the hygromycin resistance gene. The forward primer 5′‐ACTCACCGCGACGTCTGTCG‐3′ and the reverse primer 5′GCGCGTCTGCTGCTCCATA′3 were used to amplify a 727‐bp fragment of the *hpt* gene (Vain *et al*., 2003). The PCR conditions were 95 °C for 60 s, 39 cycles of 95 °C for 60 s, 63 °C for 40 s, 72 °C for 60 s. After the last cycle, the reaction was subjected to 72 °C for 6 min.

For the analysis of T_1_‐progeny, forward primers annealed in the 35S promoter and reverse primers were located in the coding region of *PAPhy_a* (Figure 1a). The following primers were used to amplify a 882 bp fragment: forward: 5′CTGACGTAAGGGATGACGCA′3, reverse 5′GCTGGTAGGTCTCGTGGATG′3. PCR conditions were 95 °C for 2 min, then 34 cycles of 95 °C for 30 s, 63 °C for 40 s, 72 °C for 40 s and then 72 °C for 2 min.

The transgene in T_2_‐progenies was verified by PCR analysis utilizing the primers for the hygromycin resistance gene described above.

### Genomic DNA gel blot analysis of T_1_‐progeny

Genomic DNA (10.0 μg) was digested overnight with *BamH*I and separated in a 1% gel. After transfer to Hybond N+, the membrane was hybridized with probes labelled with [^32^P]. The probe was a 420‐bp fragment of the hygromycin resistance gene (*Hpt*) released by the digestion of pVec8GFP (Murray *et al*., 2004) with *Bam*HI and *Pst*I. The probe was labelled by Ready‐To‐Go DNA‐labelling beads (Amersham Biosciences). Transfer and hybridization was performed as described by Holme *et al*. (2006). *BamH*I cleaves the vectors upstream the *Hpt* gene, releasing the *Hpt* intact with a minimum size of 2150 bp to be recognized by the probe (Figure 1).

### Analysis of phytase activity

The phytase activities in grains of T_0_‐, T_1_‐ and T_2_‐plants were analysed in milled flour from randomly selected samples of 20–25 grains from each transformant. The phytase activities in green flag leaves of T_2_‐plants were analysed in grinded leaves. Mature dry leaves, stems, rachis, chaff and awns utilized for phytase assays were freshly harvested or stored for 3 years at room temperature. Prior to analysis, the tissues were milled to fine powder in an IKA TUBE Mill. Protein extraction, assaying and incubation for 1 h were performed as described already (Brinch‐Pedersen *et al*., 2000; Engelen *et al*., 1994). Soluble inorganic phosphate (Pi) present in the extract already before phytase assaying was quantified by adding molybdovanadate reagent to aliquots of the soluble protein extracts before incubation. The Pi content of this extract was subtracted from the Pi quantified after assaying the phytase activity. The final value appearing after this subtraction represents only the Pi released by the phytase during incubation. The phytase activity of all material was determined in four repetitions.

### Samples preparation for nanoLC‐MS/MS

Flag leaves from nontransformed Golden Promise and *35S:PAPhy_a‐*28.4 were collected just before the appearance of the awns and frozen in liquid nitrogen. One hundred milligram of leaf material was homogenized in 1 mL of extraction buffer containing 25 mm Tris‐HCl buffer, pH 8.0, 200 mm Mannitol, 10 mm ascorbic acid, 2 mm EDTA and 0.4% (v/v) protease and phosphatase inhibitor cocktail (Sigma P8849 and P0044) by the help of liquid nitrogen in a mortar. The homogenate was centrifuged for 5 min at 20 000 ***g***, and the supernatant was used for proteomics. Sample preparation for proteomics analysis was performed as described in Christensen *et al*. (2014).

### NanoLC‐MS/MS

A nanoflow UHPLC instrument system (Easy nLCDionex Ultimate 3000 Rapid separation (RSLC) nano, Proxeon Biosystems) was coupled online to a Q‐Exactive mass spectrometer (Thermo Fisher Scientific) by a Z‐spray nanoelectrospray ion source. Chromatography column was packed in‐house with ReproSil‐Pur C18‐AQ 3‐μm resin (Dr. Maisch GmbH) in buffer A (0.5% acetic acid). The peptide mixture (0.5 μg) was loaded (3 μL) onto a C18‐reversed phase column (50 cm long, 75 μm inner diameter) and separated with a linear gradient of 3%–40% buffer B (100% acetonitrile and 0.1% formic acid) at a flow rate of 250 nL/min controlled by IntelliFlow technology over 210 min. MS data were acquired using a data‐dependent acquisition (DDA) Top10 method dynamically choosing the most abundant precursor ions from the survey scan (400–2000 m/z) for HCD fragmentation. Survey scans were acquired at a resolution of 70 000 at m/z 200, and resolution for HCD spectra was set to 17 500 at m/z 200. Normalized collision energy was 30 eV, while the underfill ratio was 0.1%. Data analysis was performed by Proteome Discoverer 1.4 (ThermoScientific). The proteome discoverer search workflow included a filter for raw file mass deisotope and charge deconvolution, a trypsin/chymotrypsin search into the integrated Sequest algorithm using 10 ppm peptide tolerance and 0.2 Da fragment tollerance, carbamidomethylation C (fixed) and as variable carbamylation N‐term, oxidation MWHPKDWFRY, dioxidation WFR, formyl (N‐term), ethanolamine (N‐term), acetyl (N‐term), deamidation NQ, methylation, proline oxidation to pyrrolidinone and phosphorylation STY. Automatic annotation was downloaded from the Proxeon web server and validation of phosphorylation sites with PhosphoRS algorithm. *Hordeum vulgare* proteins have been retrieved as Swissprot/TrEMBL (www.uniprot.org) fasta file and used as protein sequence search database. Protein annotation has been performed by Blast2GO tool (Conesa *et al*., 2008) and further manually blasted in GenBank for extra annotation/curation of the selected proteins. For absolute relative quantification, 50 fmoles/μL of yeast enolase 1 (Uniprot accession number P00924) tryptic digest was spiked to each sample. Relative quantification to ENO1 was performed by Proteome Discoverer protein score and taking into account unique peptides related to different isoforms of the proteins. Peptide mapping was performed by Peptide Finder ver 2.0 (Thermo Fisher Scientific). All determinations were performed in triplicates on tree biological samples.

### Statistics

The Welch's *t*‐test for unequal variances was employed in all phytase activity experiments to test significant differences in phytase activity between the nontransformed control plants and the transformants.

## Conflict of interests

The authors declare no conflict of interests.

## Supporting information


**Figure S1** PCR analysis of T_1_‐plants from plants transformed with *35S:PAPhy_a*.
**Figure S2** Southern blot analysis of T_1_‐plants from plants transformed with *35S:PAPhy_a*.
**Figure S3** Tryptic peptide mapping of the PAPhy_a protein.
