# Supplementary material for: Barley HvPAPhy_a as transgene provides high and stable phytase activities in mature barley straw and in grains
Source: Plant Biotechnol J. 2016 Nov 1;15(4):415–22. doi: 10.1111/pbi.12636 (PMC5362685; doi:10.1111/pbi.12636)
Supplement: Supplementary file 1 — Figure S1 PCR analysis of T1‐plants from plants transformed with 35S:PAPhy_a. Figure S2 Southern blot analysis of T1‐plants from plants transformed with 35S:PAPhy_a. Figure S3 Tryptic peptide mapping of the PAPhy_a protein. [file PBI-15-415-s001.docx]

**Supporting i nformation**

**GP 10 5 16 6 9 28**

2 4 1 3 1 2 1 3 1 3 4 1 4

**bp**

**1000**

**750**


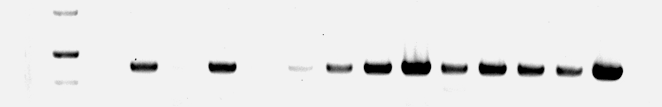


**Figure S1.** PCR analysis of T_1_-plants from plants transformed with *35S:PAPhy_a*. The PCR was performed with a forward primer located in the 35S-promoter and reverse primers located in the cDNA of *PAPhy_a*. Lane 1: non-transformed Golden Promise, lanes 2–13 , T_1_ progeny with bold numbers corresponding to the T_0_ numbers in Figure 2 and numbers underneath corresponding to the progeny number.

**Figure S2.** Southern blot analysis of T_1_-plants from plants transformed with *35S:PAPhy_a* . The probe was a 420 bp-fragment of the *HPT*-gene. Lane 1: non-transformed Golden Promise, lanes 2–5, *35S:PAPhy_a* T_1_-lines 5.1; 6.1; 9.4; 28.4.


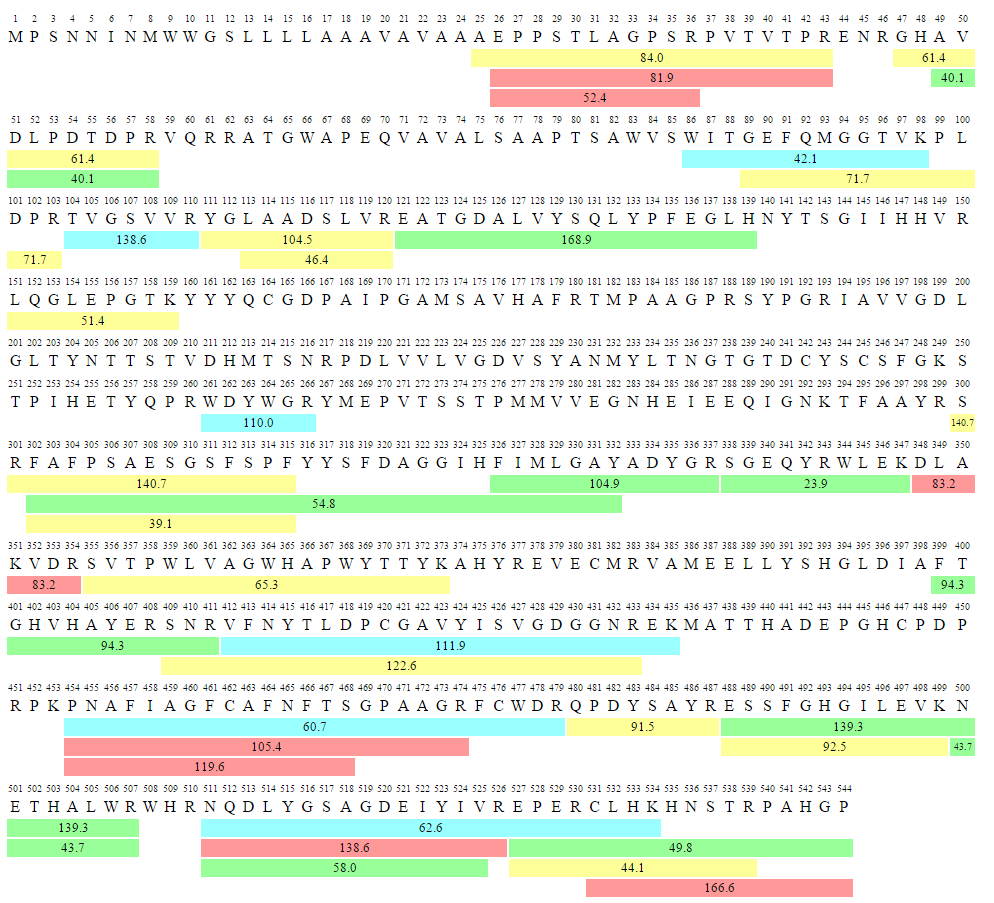


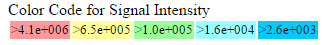


**Figure S3.** Tryptic peptide mapping of the PAPhy_a protein (C4PKL2) as results from the search into the total soluble leaf proteins of golden promise 35S::PAPhy_a (line 28.4) using Peptide Finder v. 2.0 (ThermoScientific, USA). Protein coverage calculated of two biological samples in triplicate was 72% ± 2.45. The peptide mapping shown here is from the sample with highest protein coverage.
